# Looking Inwards: The Role of Self‐Care, Self‐Management and Self‐Healing in Musculoskeletal Pain

**DOI:** 10.1002/msc.70169

**Published:** 2025-07-25

**Authors:** Joyce McSwan, Catherine E. Panwar, Ali Mobasheri

**Affiliations:** ^1^ PainWISE Pty Ltd Gold Coast Australia; ^2^ Panwar Health Pty Ltd Wamberal Australia; ^3^ Research Unit of Health Sciences and Technology Physics and Technology University of Oulu Oulu Finland; ^4^ Department of Regenerative Medicine State Research Institute Centre for Innovative Medicine Vilnius Lithuania; ^5^ Department of Joint Surgery Sun Yat‐sen University Guangzhou China; ^6^ Faculté de Médecine Université de Liège Liège Belgium

**Keywords:** integrative, musculoskeletal, pain, self‐care, self‐healing, self‐management

## 1 Introduction

Chronic musculoskeletal (MSK) pain is a significant and growing public health concern with a profound impact on individuals and society. With an estimated 149 million years of living with disability globally, the cost of treatment and loss of productivity has impacts at societal levels (World Health Organization 2022). A recent study focussed on patients with high body mass index noted $60.5 billion in direct healthcare costs and $120 billion in productivity losses globally (Chen et al. 2023). It is a common symptom of MSK disorders and most acute cases are expected to resolve with appropriate healing (El‐Tallawy et al. 2021). However, an unacceptably large proportion (up to 50%) transitions into chronic pain, a condition in its own right (Alkassabi et al. 2022; Fayaz et al. 2016). Chronic MSK pain is not only physically disabling but also contributes to psychological distress and considerable economic burden through lost productivity and healthcare costs (El‐Tallawy et al. 2021). Current pharmacological and surgical treatment options often fall short, either failing to provide long‐term relief or introducing undesirable side effects, leaving a critical need for more effective and sustainable solutions (El‐Tallawy et al. 2021).

## 2 The Intricately Interwoven Nature of Chronic MSK Pain

Steps forward are possible, as our understanding of pain has shifted from a purely biological perspective to a more nuanced and multidimensional view. Traditionally, the biomedical model dominated clinical thinking, positing that pain was primarily a consequence of identifiable tissue damage or structural abnormalities. However, this model has proven insufficient, with evidence revealing a poor correlation between imaging findings (such as degenerative changes) and a patient's reported pain severity or functional limitations (Smart 2023). This disconnect underscores a critical need to move beyond pathology alone to explain the lived experience of chronic pain. Additionally, much like the reliance on imaging to guide action, a heavy singular reliance on pharmacological pain relief and a poor understanding of pain may contribute to the helpless cycle of pain (Moretti et al. 2024).

**FIGURE 1:**
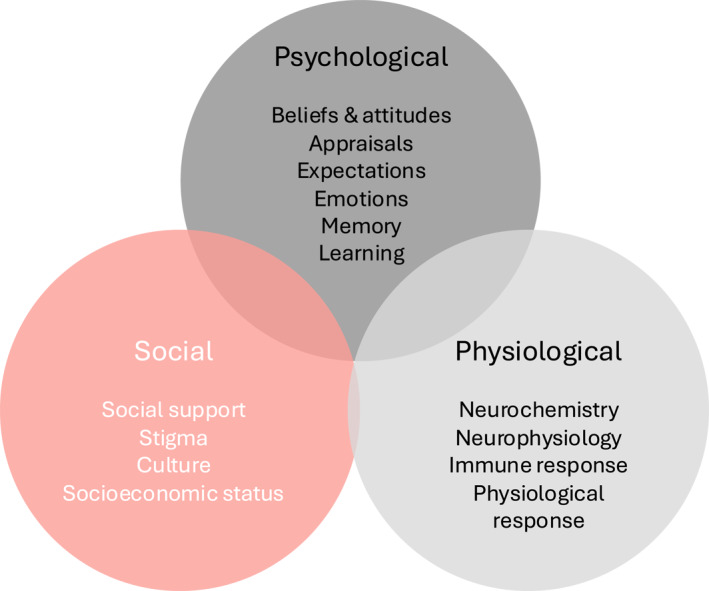
The biopsychosocial model of pain.

The biopsychosocial model now forms the foundation for modern pain science (Figure 1). This framework recognises that chronic pain is not just a symptom of physical injury. It arises from a complex interplay of biological, psychological, and social influences, each of which may be shaped by genetic predisposition alongside social and environmental exposures (Dunn et al. 2024; Smart 2023). Central and peripheral sensitisation often underpin seemingly aberrant responses to the stimulation of nociceptive neurons. These responses include hyperalgesia, allodynia, temporal summation of pain, and diffuse noxious inhibitory control (Smith et al. 2019). On a biological level, evidence implicates mechanisms such as microvascular dysfunction (Coderre 2023), neuroinflammation in both central and peripheral nervous systems (Fang et al. 2023), and changes in brain structure and function (neuroplasticity) (De Ridder et al. 2021; Pelletier et al. 2015).

Psychological and emotional factors are also central to chronic pain vulnerability and expression. Negative effect, including anxiety and depression, is consistently associated with reduced pain tolerance and amplification of pain in chronic conditions (Frumkin and Rodebaugh 2021). Attentional focus and emotional responses, whether positive or negative, can lead to maladaptive behaviours that also reinforce the pain cycle (Amaro‐Diaz et al. 2022; Yarns et al. 2022). Catastrophising, including persistent fear of pain, perceived injustice, and low self‐efficacy, is particularly detrimental (Anselmo et al. 2024; Kardash et al. 2024; Reme et al. 2022). These cognitive distortions often accompany physiological changes, such as autonomic nervous system dysregulation and general pathophysiology and dysregulation of the hypothalamic‐pituitary‐adrenal (HPA) axis, resulting in stress‐induced hyperalgesia (Wyns et al. 2023). So too, disrupted sleep, circadian rhythm and pharmacological influences modulate pain perception and persistence (Daguet et al. 2022; Runge et al. 2024).

**FIGURE 2:**
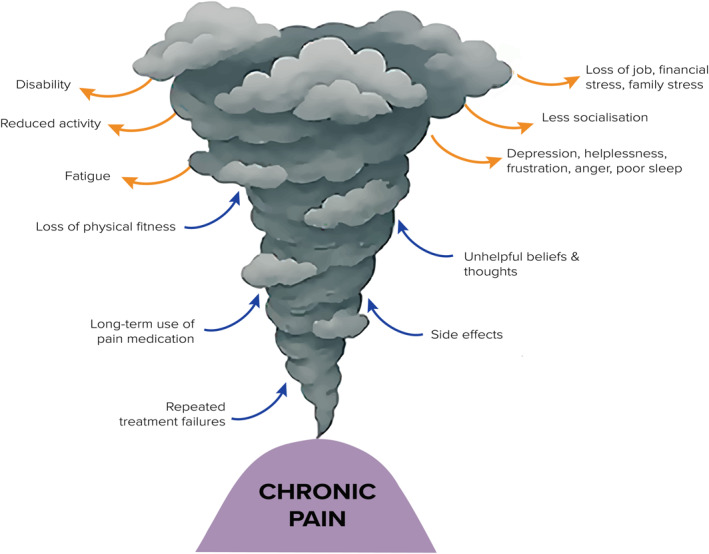
Causes of excessive distress and suffering in chronic pain.

Social and environmental contexts also shape the chronic pain experience. Factors such as cultural beliefs, prior pain experiences, socioeconomic status, employment, living conditions, substance use disorders, and language barriers can influence both vulnerability to pain and resilience in the face of it (Kapos et al. 2024). Significantly, stigma and invalidation by society, healthcare professionals, or health systems and insurers can further exacerbate the chronic pain burden (Perugino et al. 2022). Patients often report feeling disbelieved or dismissed, notably when their pain lacks a clear cause (Nicola et al. 2022). This societal invalidation can lead to reduced help‐seeking, increased emotional distress, and poor engagement in care pathways (Nicola et al. 2022). Moreover, these social determinants may exert varying influence throughout life, highlighting the importance of personalised, developmentally appropriate pain management strategies. Addressing stigma and ensuring validation of the pain experience are essential steps in fostering therapeutic relationships, improving pain management adherence, and supporting effective self‐management. Together, these and other factors influence the impact and disability of chronic pain (Figure 2).

## 3 Self‐Care and Self‐Management Are Person‐Centred Components of the Preservation of Health

**FIGURE 3:**
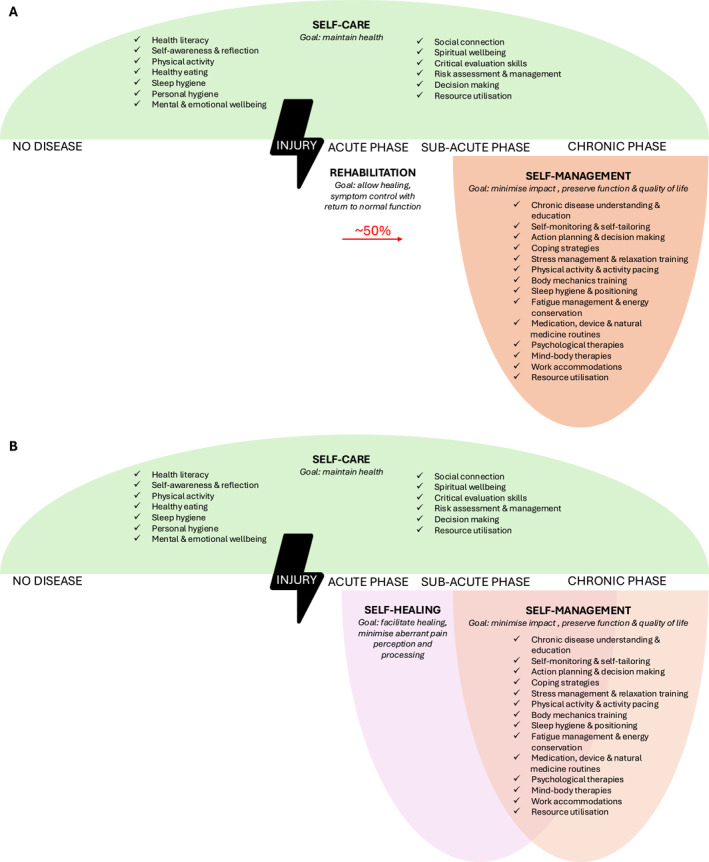
(A) The interwoven concepts of self‐care and self‐management and (B) place of self‐healing in an evolved MSK pain management landscape, facilitated by self‐care and self‐management interventions.

Despite high rates of healthcare utilisation, most suffering associated with MSK conditions occurs in daily life, outside interactions with formal health systems (Gron et al. 2025). This highlights the importance of actively empowering individuals to participate in their health between clinical visits, and there is a growing recognition for the role of person‐centred strategies to be at the heart of MSK pain management. Self‐care and self‐management are labels given to two such aspects, and while deeply intertwined, they have distinct goals (Figure 3A). Both are enacted by individuals in their everyday lives and work together to promote function, healing, and resilience and are underpinned by knowledge, skills, social influences, attitude, and self‐efficacy (Kongsted et al. 2021). Self‐care encompasses the broad range of early corrective actions that individuals take to stay healthy and prevent disease. This includes building health literacy, maintaining mental wellbeing and self‐awareness, engaging in regular physical activity, eating healthily, avoiding health risks, practising good hygiene, and appropriately using medications or diagnostic tools (International Self‐care Foundation 2025; Kovacevic et al. 2018). These behaviours not only support overall wellbeing but can buffer against the exacerbation of chronic conditions.

Self‐management, on the other hand, is a specific subset of self‐care that focuses on dealing with chronic illness once symptoms and aberrant pathways have set in. It refers to an individual's ability to actively engage in strategies that help manage symptoms, maintain function, prevent disease progression, and improve quality of life (Kongsted et al. 2021; McSwan et al. 2021). In musculoskeletal pain, this may include using natural remedies, adopting targeted nutrition plans, applying therapeutic devices, learning and practising psychological coping strategies, goal setting for and pacing of physical activity, optimising mobility, and improving sleep hygiene (Shi and Wu 2023).

However, during the post‐injury rehabilitation window in the acute phase, there has been a lack of defined strategies to facilitate healing. Particularly, those that lean into the body's innate repair mechanisms that may assist in reducing the unacceptably high rate of transition to chronic pain.

Although the current biopsychosocial models of pain care have increased the number of tools available to manage MSK pain, there remains a suboptimal impact on pain chronicity (Fayaz et al. 2016). A gap between health‐promoting self‐care behaviours in the absence of injury and the initiation of self‐management strategies when chronic pain has set in presents an untapped window of opportunity. In the subacute phase (up to 12 weeks post injury), it may be possible to optimise recovery and healing and preserve health through activation of some of the body's innate repair mechanisms.

## 4 Defining Self‐Healing as an Additional Goal of Care in MSK Pain Management

An emerging understanding in pain science suggests that relief from MSK pain may not solely depend on external interventions but may also be achieved by supporting and enhancing the body's innate self‐repair mechanisms. The concept of self‐healing reframes pain management by recognising the dynamic role of endogenous processes that, when optimally functioning, can restore equilibrium and contribute to recovery (McSwan et al. 2021). Specifically, pain disrupts several interconnected body systems, and healing may hinge on re‐establishing balance across five core physiological and psychological domains: circulation, nervous system regulation, muscular tension, inflammation, and psychological coping (McSwan et al. 2021; Mobasheri 2022). By activating these systems early, during the rehabilitation phase post‐injury, self‐healing may enhance existing rehabilitation strategies to not just allow but facilitate normal tissue healing, and not just control pain but rather minimise aberrant pain perception and processing (Figure 3B).

Specifically, adequate circulation is central to tissue repair and pain relief. Enhancing microcirculation and promoting vasodilation allows for improved delivery of oxygen and nutrients, facilitating the healing of damaged tissues (McSwan et al. 2021). In parallel, activating the parasympathetic nervous system, often suppressed in chronic stress and pain states, can reduce the heightened sympathetic arousal perpetuating pain (Nazarewicz et al. 2015; Tindle and Tadi 2025). Muscular tension, another hallmark of chronic pain, restricts blood flow and exacerbates discomfort. The body can restore metabolic exchange and decrease nociceptive input by encouraging muscle relaxation. Inflammation plays a dual role: while it is essential for initiating tissue repair, persistent or unresolved inflammation can maintain pain long after structural healing has occurred (Gallo et al. 2017). Supporting the timely resolution of inflammation, rather than simply suppressing it, may be a key to achieving long‐term pain relief. Finally, psychological coping mechanisms, including emotional regulation and cognitive patterns such as catastrophising, profoundly influence the pain experience (Edwards et al. 2016). Addressing these maladaptive behaviours is critical to breaking the chronic pain cycle and fostering resilience.

An integrated, multimodal approach is essential to support the body's self‐healing potential (McSwan et al. 2021; Mobasheri 2022). This may include interventions to optimise inflammatory responses, enhance tissue regeneration, facilitate recovery, and break the vicious cycle of pain to return to normal, daily function. These include circadian regulation and sleep restoration, dietary immunomodulation and photobiomodulation, along with early mobilisation with graded corrective movement techniques and build functional capacity through graded techniques and load management (Al Balah et al. 2025; Field et al. 2024; Ham and Kim 2025; Klyne and Hall 2024; Merkle et al. 2020; Warfield et al. 2021). At a cognitive processing level, general stress and vagus nerve stimulation can reduce sympathetic overactivation with controlled breathing techniques (Shao et al. 2023), while at the tissue level, thermotherapy, contrast hydrotherapy and manual lymphatic drainage can help to maintain the balance of extracellular fluids, facilitating tissue perfusion and egress of waste products that sensitise nociceptors and perpetuate pain (Mooventhan and Nivethitha 2014; Tuckey et al. 2021). At a neurobiological level, sensorimotor recalibration using mirror therapy and other techniques, such as acupuncture and mind‐body movement (Langevin 2021; Liu et al. 2017), is being used to promote neuroplasticity and enhance neuromuscular control (Nishi et al. 2024).

Exercise can activate endogenous hypoalgesic responses and may be used to recondition pain modulation response pathways without exacerbating underlying pathology (Smith et al. 2019; Tanaka et al. 2023). This necessitates graded physiotherapy expertise to incorporate mobility, muscle strength, balance, and proper biomechanics and force distribution. Not all patients may be able to tolerate these evidence‐based therapies; therefore, it is important that a personalised approach is taken alongside a variety of non‐drug interventions. One such intervention is gait modification, which is a growing treatment approach to optimise specific function components by modifying spatiotemporal, kinematic, or joint load variables. These strategies rely on motor learning and skill acquisition to ensure integration into habitual movement patterns and enhance long‐term adherence (Charlton et al. 2021). Another is electromyostimulation, which has shown that stimulation of muscle activity and improvement of neuromuscular coordination without overloading painful joints can result in significant pain reduction and improvement in physical function and mobility in people with knee osteoarthritis (Kast et al. 2024).

Another emerging area of pain science is gastrointestinal health, specifically the role of the gut microbiome in modulating systemic inflammation, immune signalling, and neurological processing related to pain perception (Alkassabi et al. 2022). Several mechanisms have been proposed where the gut microbiome may influence musculoskeletal health. Microbial metabolites, including short‐chain fatty acids, lipopolysaccharide, and trimethylamine N‐oxide, are understood to regulate inflammation, bone metabolism, and muscle function, with dysbiosis directly affecting inflammation and oxidative stress (La Placa et al. 2025). Interventions that target the microbiome‐gut‐immune brain axis, such as prebiotics, probiotics, synbiotics, postbiotics, whole diet balance, fermented foods, physical activity, and faecal microbiota transplantation, are strategies under investigation (O'Riordan et al. 2025).

At the same time, psychological and mindfulness interventions, such as cognitive behavioural therapy (CBT), acceptance and commitment therapy (ACT), pain reprocessing therapy (PRT), and mindfulness meditation, help individuals reframe their pain experience, reduce emotional distress, and build adaptive coping skills (Wang et al. 2025).

While pharmacological treatments still play an important role in pain management, their most significant value may be enabling patients to engage in broader self‐regulation strategies rather than serving as the sole or primary means of symptom relief. Holistic and integrative perspectives hold promise for reducing pain and empowering individuals to take an active role in their recovery, fostering sustainable improvements in function and well‐being. It provides another opportunity for patients to take a lead role in their recovery as they self‐observe, self‐regulate and develop sustainable behaviour change before chronic pain sets in.

## 5 Summary

The concept of self‐healing post MSK injury has the potential to offer a paradigm shift in rehabilitation, and if implemented early, it could be a chance to change a patient's trajectory towards chronic pain. With a whole‐body lens and through mastery of environmental, functional, mental and lifestyle influences from the early onset of tissue healing, pain perception and processing, we may be able to extend the current focus towards recovery and regeneration and from pain freedom to improving the body's adaptive reserve with targeted self‐healing interventions in the subacute period.

## Author Contributions

JM, CP and AM all equally contributed to conceptualisation, writing the original draft, and reviewing and editing the work.

## Conflicts of Interest

CP has been a paid consultant for PainWISE Pty Ltd, Reckitt Benckiser and iNova Pharmaceuticals.

## Data Availability

Data sharing is not applicable to this article as no new data were created or analysed in this study.
